# Rocky Mountain Spotted Fever Mimicking Multisystem Inflammatory Syndrome in Hospitalized Children, Sonora, Mexico

**DOI:** 10.3201/eid3007.240033

**Published:** 2024-07

**Authors:** Gerardo Álvarez-Hernández, Cristian N. Rivera-Rosas, J.R. Tadeo Calleja-López, David W. McCormick, Christopher D. Paddock, Jehan Bonizú Álvarez-Meza, Fabián Correa-Morales

**Affiliations:** University of Sonora, Hermosillo, Mexico (G. Álvarez-Hernández, C.N. Rivera-Rosas, J.R.T. Calleja-López);; Centers for Disease Control and Prevention, Atlanta, Georgia, USA (D.W. McCormick, C.D. Paddock);; Sonora Children’s Hospital, Hermosillo (J.B. Álvarez-Meza);; Centro Nacional de Programas Preventivos y Control de Enfermedades, Mexico City, Mexico (F. Correa-Morales)

**Keywords:** Rocky Mountain spotted fever, *Rickettsia*, multisystem inflammatory syndrome, children, MIS-C, COVID-19, respiratory infections, severe acute respiratory syndrome coronavirus 2, SARS-CoV-2, SARS, coronavirus disease, viruses, coronavirus, bacteria, vector-borne infections, zoonoses, Mexico

## Abstract

We describe 5 children who had Rocky Mountain spotted fever (RMSF) and manifested clinical symptoms similar to multisystem inflammatory syndrome in Sonora, Mexico, where RMSF is hyperendemic. Physicians should consider RMSF in differential diagnoses of hospitalized patients with multisystem inflammatory syndrome to prevent illness and death caused by rickettsial disease.

Rocky Mountain spotted fever (RMSF), a tickborne disease caused by *Rickettsia rickettsii*, is the leading cause of death from rickettsial infections in the Western Hemisphere (*1*). The disease can progress rapidly to death or severe illness in persons who do not receive appropriate antimicrobial drug therapy within the first 5 days after illness onset (*1*–*3*).

Hyperendemic RMSF levels are present in several communities in the southwestern United States and multiple states across northern Mexico (*4*). Case-fatality rates in northern Mexico are 27%–33% (*5*,*6*). A total of 2,176 RMSF cases were identified in Mexico during 2015–2023, of which 933 (42.9%) patients were children and adolescents 1 month–19 years of age (*7*). Sonora, in northwestern Mexico, has the greatest RMSF burden within the country; 222 pediatric cases were identified during 2015–2022, comprising ≈50% of the state’s total RMSF incidence during that period (*6*).

During March 2020–December 31, 2022, a total of 16,207 pediatric cases of COVID-19 occurred in Sonora, of which 434 (2.7%) case-patients were hospitalized and 36 died (case-fatality rate 0.22%) (*8*). However, national- or state-level data are not available for cases of multisystem inflammatory syndrome in children (MIS-C). The early signs and symptoms of RMSF are nonspecific and include fever, headache, rash, nausea, vomiting, arthralgia, and myalgia. Early manifestations of RMSF often resemble other common pediatric febrile illnesses, such as Kawasaki disease and toxic shock syndrome (*1*–*3*). As RMSF progresses, abdominal pain, extremity edema, dyspnea, tachycardia, hypotension, organ edema, hemorrhages, septic shock, distal necrosis of the extremities, and death can occur (*3*,*9*). The clinical profile of MIS-C (*10*,*11*) overlaps with that of severe rickettsioses, particularly during the later stages of RMSF infections, and this clinical similarity might lead to critical gaps in prompt diagnosis and treatment of both diseases in endemic regions. We describe 5 pediatric patients with RMSF admitted to a hospital in Sonora who were initially suspected of having MIS-C.

## The Study

We identified patients after reviewing 8,432 inpatient admissions at the Sonora Children’s Hospital in Hermosillo, Mexico, during September 30, 2020–December 31, 2022. During the study period, RMSF was diagnosed in 61 patients, COVID-19 in 27 patients, and MIS-C in 13/27 (48%) patients who had COVID-19 (*12*). We retrieved sociodemographic and clinical characteristics from medical charts. The study was approved by the Sonora Children’s Hospital Institutional Review Board.

We used the World Health Organization case definition to identify patients with MIS-C (*13*); we defined evidence of COVID-19 as detectable SARS-CoV-2 antibodies in serum samples or a positive antigen test from a nasopharyngeal swab sample. We defined an RMSF case as a patient who had >2 acute clinical manifestations within 7 days, such as fever, headache, malaise, rash, diarrhea, vomiting, and a history of tick exposure, along with a single positive *Rickettsia* confirmatory test. Confirmatory tests included detection of *R. rickettsii* or *Rickettsia* spp. DNA in whole-blood specimens by using quantitative PCR (qPCR) or detection of *R. rickettsia*–specific IgG at a titer of >1:256 in a single serum specimen by using an indirect immunofluorescence assay.

We identified 5 patients with RMSF who also met World Health Organization criteria for MIS-C. The median patient age was 8 (range 6–17) years; 4 were male and 1 female (Table 1). The median duration from illness onset to hospital admission was 10 (range 4–10) days. All patients had a fever and rash; other common symptoms were abdominal pain in 3 (60%) patients and edema of the hands and feet in 3 (60%) patients. The rash preceded hospital admission in 4 (80%) patients. All patients reported a history of tick exposure and received corticosteroids; 3 received intravenous immunoglobulin for treatment of suspected MIS-C.

**Table 1:** Demographic, clinical, and laboratory test characteristics of patients at hospital admission in study of Rocky Mountain spotted fever mimicking multisystem inflammatory syndrome in hospitalized children, Sonora, Mexico*

Variable	Reference range	Case 1	Case 2	Case 3	Case 4	Case 5
Age, y		6.2	17.9	11.0	7.1	8.7
Sex		M	M	F	M	M
BMI Z-score†		0–1	1–2	1–2	1–2	>2
Laboratory confirmation of SARS-CoV-2 infection‡		Antigen+, IgM+	IgG+	IgG+, IgM+	IgG+	IgG+
Laboratory confirmation of *Rickettsia rickettsii* infection		IFA IgG 1:256	PCR	PCR	IFA IgG 1:2,048	PCR,* Rickettsia* spp.
Leukocyte count, × 10^3^/μL	4.6–10.2	4.3	6.8	15.7	10.5	19.9
Platelet count, × 10^3^/μL	150–450	72.0	57.0	45.0	22.0	317.0
Procalcitonin, ng/mL	0–0.5	10.8	2.6	75.4	6.6	0.8
C-Reactive protein, mg/dL	<0.5	10.8	17.1	10.2	6.6	NA
D-dimer, μg/mL	0–0.5	17.6	NA	7.9	3.6	NA
Ferritin, ng/mL	21–274	1,099.8	NA	5,474.4	3,229.2	1,260.30
Prothrombin time, s	11.1–14.1	14.7	16.0	14.0	17.6	16.3
Fibrinogen, mg/dL	200–400	215.0	NA	134.0	329.0	379.0
ESR, mm/h	3–10	35.0	26.0	20.0	65.0	40.0
LDH, U/L	240–480	NA	738.0	754.0	880.0	569.0
AST, U/L	5–34	91.0	186.0	111.0	121.0	69.0
ALT, U/L	0–55	44.0	76.0	47.0	54.0	77.0

*ALT, alanine aminotransferase; AST, aspartate aminotransferase; BMI, body mass index; ESR, erythrocyte sedimentation rate; IFA, indirect immunofluorescence assay; LDH, lactate dehydrogenase; NA, not available; ; +, positive. †Calculated by using data from the World Health Organization (https://www.who.int/tools/growth-reference-data-for-5to19-years/indicators/bmi-for-age). According to those data, a normal BMI is defined as a Z-score of −1 to 1, an overweight BMI is defined as a Z-score of 1–2, and an obese BMI is defined as a Z-score of >2. ‡Confirmed by presence of SARS-CoV-2–specific IgG or IgM in serum samples or a positive antigen test from a nasopharyngeal swab sample.

*R. rickettsii* infection was confirmed by IFA for 2 patients whose blood samples were drawn on the 6th and 13th days after onset of symptoms. Two patients were positive for *R. rickettsii* by qPCR, and qPCR detected *Rickettsia* spp. in 1 case. Although all patients received their first medical consultation within 1–3 days after onset of symptoms, a delay in clinical suspicion of RMSF beyond day 5 was found in 4 (80%) cases; the median delay in prescribing specific antimicrobial drugs for RMSF was 10 (range 3–13) days after symptom onset (Table 2). Doxycycline treatment was belatedly initiated in all 5 patients, in 4 at the time of hospital admission and in 1 patient 3 days after hospitalization.

**Table 2:** Medical therapies and selected characteristics of patients in study of Rocky Mountain spotted fever mimicking multisystem inflammatory syndrome in hospitalized children, Sonora, Mexico*

Case no.	IVIg	Corticosteroid	Anticoagulation prophylaxis	Vasopressor or inotrope	First outpatient medical consultation, d†	Treatment delay, d‡	Hospital care, d
1	1 g/kg, day 1; 2 g/kg, day 2	Methylprednisolone IV, 30 mg/kg/dose; dexamethasone IV, 8 mg/d	Enoxaparin, 0.5 mg/kg/dose	Dobutamine, 5 µg/kg/min; norepinephrine, 0.05 µg/kg/min	1	13	15
2	NA	Hydrocortisone IV, 4 mg/kg/d; methylprednisolone IV, 1 g/d	NA	Dobutamine, 5 µg/kg/min; norepinephrine, 0.1 µg/kg/min	1	6	4
3	1 g/kg/day for 2 d	Methylprednisolone IV, 1 mg/kg/d; dexamethasone IV, 0.2 mg/kg/dose; hydrocortisone IV, 2 mg/kg/dose	Enoxaparin SC, 0.5 mg/kg/dose; aspirin by mouth, 60 mg/kg/d	Norepinephrine, 0.22 µg/kg/min	3	3	15
4	NA	Hydrocortisone IV, 1 mg/kg/dose	NA	Norepinephrine, 0.22 µg/kg/min; dobutamine, 8.3 µg/kg/min	1	10	13
5	1 g/kg/day for 2 d	Methylprednisolone IV, 1 mg/kg/d; prednisone PO, 1 mg/kg/d	Enoxaparin SC, 0.5 mg/kg/dose	NA	3	10	4

*IV, intravenous; IVIg, intravenous immunoglobulin; NA, not applicable; SC, subcutaneous. †Number of days until first outpatient medical consultation after symptom onset. ‡Delay in doxycycline treatment after symptom onset; doxycycline was initiated at the time RMSF was clinically suspected during the hospital stay.

Laboratory test abnormalities seen in the 5 patients included thrombocytopenia; elevated inflammatory markers such as C-reactive protein, procalcitonin, D-dimer, ferritin, and aminotransferases; and leukocytosis (Table 1). Four patients were admitted to the hospital’s intensive care unit and required vasopressor support. Echocardiography was performed for 3 patients: 1 patient had no abnormal findings, 1 patient had trace pericardial effusion, and 1 patient had a 0.5 cm pericardial effusion and tricuspid regurgitation. The median duration of hospitalization was 13 (range 3–15) days. No patients died, and severe sequelae were not reported.

## Conclusions

RMSF and MIS-C share clinical and laboratory test abnormalities and distinguishing between those 2 conditions can be challenging for clinicians in endemic regions. We describe 5 children who had clinical and laboratory evidence of both RMSF and MIS-C. Those patients improved with doxycycline treatment, highlighting the importance of timely recognition and treatment of RMSF, even when clinicians suspect SARS-CoV-2 infections. Because those infections can co-exist, a high degree of clinical suspicion is necessary. A history of tick exposure should prompt clinicians to consider RMSF, although tick bites are recognized in <50% of RMSF cases.

Clinical suspicion of MIS-C should not preclude consideration of RMSF and other rickettsial diseases (*12*,*13*). Fever, rash, gastrointestinal manifestations, and abdominal pain are among the most frequent clinical features in hospitalized children with MIS-C (*10*) or RMSF (*2*,*3*,*9*). Although ≈90% of patients with MIS-C or RMSF manifest fever, a rash is present in up to 90% of RMSF patients (*6*), compared with ≈50% of children with MIS-C (*10*,*14*). Patients with RMSF and MIS-C have similar laboratory test abnormalities that include thrombocytopenia, hyponatremia, and elevated inflammatory markers (i.e., procalcitonin and C-reactive protein) (*6*,*9*,*10*,*14*), probably because both diseases are characterized by a generalized inflammatory response, endothelial damage, and increased vascular permeability (*1*,*10*).

Doxycycline is the recommended antimicrobial drug treatment for all patients who have suspected RMSF and should be empirically initiated to reduce fatal outcomes and severe sequelae (*1*,*3*,*9*), particularly in vulnerable children living with social disadvantages (*1*,*14*). Physicians and other health personnel practicing in RMSF-endemic areas should systematically consider RMSF in the differential diagnosis of hospitalized patients who have MIS-C to reduce delays in therapy and prevent death and severe sequelae caused by this rickettsial disease.
